# A whole-genome metagenomic approach to explore the gut microbiome of inflammatory bowel disease patients in Bangladesh

**DOI:** 10.1128/mra.00738-24

**Published:** 2024-11-29

**Authors:** Md. Hasanul Banna Siam, Spencer Mark Mondol, Md. Shahriar Kabir Shakil, Md. Rafiul Islam, Israt Islam, Otun Saha, ‌‌ Amiruzzaman, Raj Datta, Toufiq Ahmed, Donald James Gomes, Md. Mizanur Rahaman

**Affiliations:** 1 Department of Microbiology, University of Dhaka, Dhaka, Bangladesh; 2 Department of Biotechnology and Genetic Engineering, Noakhali Science and Technology University, Noakhali, Bangladesh; 3 Department of Microbiology, Noakhali Science and Technology University, Noakhali, Bangladesh; 4 Department of Medicine, Sir Salimullah Medical College, Dhaka, Bangladesh; 5 Sheikh Russel National Gastroliver Institute and Hospital, Dhaka, Bangladesh; 6 Dhaka Medical College and Hospital, Dhaka, Bangladesh; 7 Department of Internal Medicine, Bangabandhu Sheikh Mujib Medical University (BSMMU), Dhaka, Bangladesh; Loyola University Chicago, Chicago, Illinois, USA

**Keywords:** inflammatory bowel disease, gut microbiome, gastrointestinal illness, *Segatella copri*

## Abstract

In this study, we analyzed the gut microbiome of four Bangladeshi inflammatory bowel disease patients, identifying *Segatella copri* as the most prevalent, followed by *Phocaeicola vulgatus*, *Bacteroides xylanisolvens*, *Bacteroides fragilis*, *Bacteroides thetaiotaomicron*, *Bacteroides ovatus*, *Bacteroides uniformis*, *Phocaeicola dorei*, and many other species. Firmicutes, Proteobacteria, Bacteroidetes, and Actinobacteria were the major phyla detected.

## ANNOUNCEMENT

**Fig 1:**
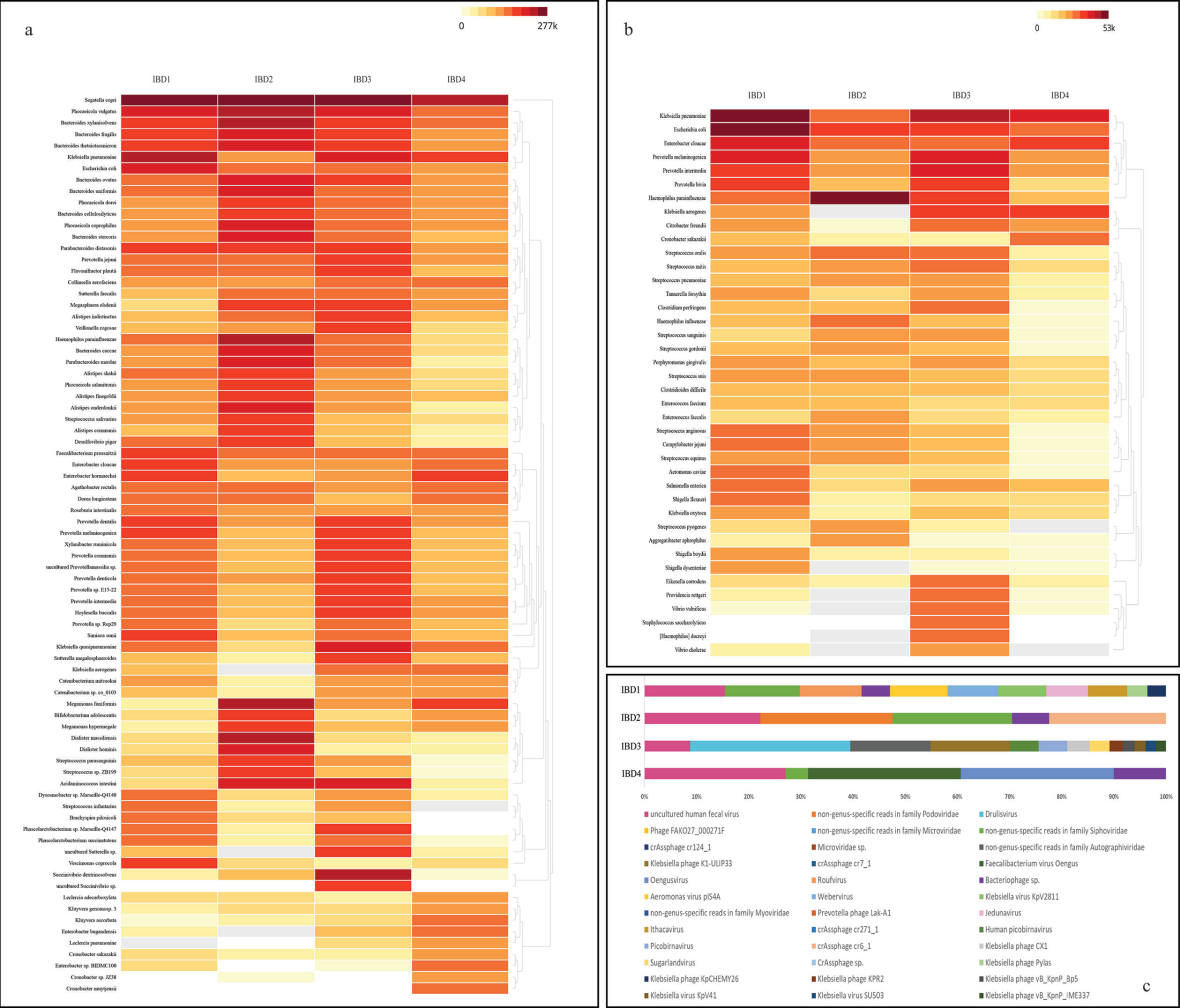
Taxonomic classification and relative abundance of the gut microbiome of IBD patients in Bangladesh. (**a**) A collective representation of the of top 30 abundant species from each sample showing the most abundant species of the gut microbiome of IBD patients in Bangladesh. (**b**) A collective representation of the most abundant pathogenic species. (**c**) Abundance of viruses in IBD patients.

Inflammatory bowel disease (IBD) is a chronic gastrointestinal illness characterized by abdominal cramps, persistent diarrhea, bloody stool, and weight loss, becoming increasingly prevalent in newly industrialized and developing countries like Bangladesh due to the adoption of a western lifestyle (1). IBD affects an estimated 6.8 million people worldwide and hospitalization rates for IBD vary depending on the subtype and the severity of the disease (2). This study aimed to explore the gut microbiome of Bangladeshi IBD patients, as no previous literature exists on this topic. We sequenced and analyzed the complete metagenome of the gut microbiota from four IBD patients from Bangladesh. The stool samples were obtained from patients diagnosed with IBD at the Sheikh Russel National Gastroliver Institute & Hospital in Mohakhali, Dhaka. QIAamp PowerFecal Pro DNA Kit (QIAGEN, Germany) was used to extract DNA from the faecal material. The concentration of genomic DNA was measured with a Qubit Fluorometer (ThermoFisher, USA), and 150 ng was used for library construction with the NEBNext Ultra II FS DNA Library Prep Kit. Libraries were PCR-amplified, cleaned, quantified, and further whole genome metagenomic shotgun sequencing was performed on an Illumina HiSeq4000 sequencer (Illumina, San Diego, CA, USA) using a 150-bp paired-end run format (300 cycle run). The sequenced raw reads were quality controlled using fastp (v0.23.4) (3). The host DNA filtration was carried out through Bowtie2 integrated within CZ ID (Formerly IDseq) (4, 5). Following quality control and host DNA filtration, the filtered reads were subjected for taxonomic classification. The taxonomic profiling process began by identifying potential bacterial, viral, and archaeal species in raw metagenomic samples using NT (NCBI Nucleotide) database from CZ ID (Formerly IDseq) (4, 5). Species abundance was calculated based on coverage and normalized by reference length through CZ ID (Formerly IDseq). Read coverage analysis was performed using Samtools (Version 1.17) (6) and Bedtools (Version 2.31.0) (7) with default parameters. After sequencing around 25–40 million reads were generated from each sample (Table 1). The average reads per sample was 33,762,426. After quality control, the average number of reads per sample was 28,973,296. The analysis of the top 30 abundant species from each sample identified a total of 79 species (Fig. 1a). The study highlighted *Segatella copri* (formerly known as *Prevotella copri*) as the most prevalent species in the gut microbiome of IBD patients. Other abundant species included *Phocaeicola vulgatus*, *Bacteroides xylanisolvens*, *Bacteroides fragilis*, *Bacteroides thetaiotaomicron*, *Bacteroides ovatus*, *Bacteroides uniformis*, and *Phocaeicola dorei*. In the gut of IBD patients, the major phyla detected were Firmicutes, Proteobacteria, Bacteroidetes, and Actinobacteria. The phyla Firmicutes, Proteobacteria, and Bacteroidetes were more abundant compared to Actinobacteria. Among the pathogenic species, *Klebsiella pneumoniae* was the most abundant, followed by *Escherichia coli*, *Enterobacter cloaceae*, *Prevotella melaninogenica*, and *Prevotella intermedia* (Fig. 1b). Several viral sequences were identified in the gut of IBD patients, with the majority being uncultured human fecal viruses (Fig. 1c). This study shed light on the previously unknown gut microbiome of Bangladeshi IBD patients and laid the foundation for further research. In the future, additional samples should be sequenced and examined to identify biomarkers and find strategies to develop evidence-based treatments for IBD.

**TABLE 1:** Metagenome information for the samples including read summaries and accessions

Sample ID	Sex	Age	Marital status	Stool type	Location coordinates	Diabetes	Hypertension	Total reads	Reads after fastp qc	Accession no.
IBD1	Male	31	Married	Mixed	23°46′34.0 ″N, 90°24′41.8 ″E	No	No	34,189,418	29,854,968	SRR25109741
IBD2	Male	32	Married	Alternating	23°46′34.0 ″N, 90°24′41.8 ″E	No	No	25,383,700	21,942,752	SRR25109740
IBD3	Female	40	Married	Mixed	23°46′34.0 ″N, 90°24′41.8 ″E	No	No	36,101,608	31,868,398	SRR25109738
IBD4	Male	24	Unmarried	Mixed	23°46′34.0 ″N, 90°24′41.8 ″E	No	No	39,374,976	32,227,066	SRR25109737

## Data Availability

The raw metagenome sequence data were submitted in NCBI under the BioProject ID PRJNA990354. The accession no. of the four samples (IBD1, IBD2, IBD3, and IBD4) are respectively SRR25109741, SRR25109740, SRR25109738 and SRR25109737.
